# Minor influence of patient education and physiotherapy interventions before total hip replacement on patient-reported outcomes: an observational study of 30,756 patients in the Swedish Hip Arthroplasty Register

**DOI:** 10.1080/17453674.2019.1605669

**Published:** 2019-04-17

**Authors:** Christopher Torisho, Maziar Mohaddes, Kristin Gustafsson, Ola Rolfson

**Affiliations:** a Department of Orthopaedics, Institute of Clinical Sciences, Sahlgrenska Academy, University of Gothenburg, Gothenburg;;; b Swedish Hip Arthroplasty Register, Gothenburg;;; c Division of Physiotherapy, Department of Medical and Health Sciences, Linköping University, Linköping, Sweden

## Abstract

Background and purpose — It is unclear whether physiotherapy interventions or patient education before total hip replacement (THR) is beneficial for patients postoperatively. Utilizing the Swedish Hip Arthroplasty Register (SHAR), we retrospectively studied the influence of preoperative self-reported exposure to physiotherapy and/or patient education on patient-reported outcomes 1 year after THR.

Patients and methods — Data covering all THRs performed in Sweden for osteoarthritis, between the years 2012 and 2015, was obtained from SHAR. There were 30,756 patients with complete data. Multiple linear regression modelling was performed with 1-year postoperative PROMs (hip pain on a visual analogue scale [VAS], with the quality of life measures EQ-5D index and EQ VAS, and surgery satisfaction VAS) as dependent variables. Self-reported physiotherapy and patient education (yes or no) were used as independent variables.

Results — Physiotherapy was associated with slightly less pain VAS (–0.7, 95% CI –1.1 to –0.3), better EQ-5D index (0.01, CI 0.00–0.01), EQ VAS (0.8, CI 0.4–1.2), and better satisfaction VAS (–0.7, CI –1.2 to –0.2). Patient education was associated with slightly better EQ-5D index (0.01, CI 0.00–0.01) and EQ VAS (0.7, CI 0.2–1.1).

Interpretation — Even though we found statistically significant differences in favor of physiotherapy and patient education, the magnitude of those were too small and inconsistent to conclude a truly positive influence. Further research is needed with more specific and demarcated physiotherapy interventions.

Physiotherapy, in the form of supervised exercise, has been shown to reduce pain and improve function as assessed with patient-reported outcome measures (PROMs) for hip OA patients (Hernandez-Molina et al. 2008, Fransen et al. 2014), including later stages of the disease (Rooks et al. 2006, Villadsen et al. 2014). Self-management for knee and hip osteoarthritis improves pain (Chodosh et al. 2005) up to 21 months after intervention (Kroon et al. 2014), although both studies questioned the clinical relevance due to limited effect size. A recent meta-analysis including 13 RCTs reported a positive effect of preoperative exercise and patient education on postoperative pain for hip OA (Moyer et al. 2017). The meta-analysis found large differences in published studies with regard to interventions and minimal reporting on confounders.

In Sweden, core treatment of OA is standardized in a national educational self-management programme for hip and knee OA patients, the Supported Osteoarthritis Self-Management Programme (SOASP) (Thorstensson et al. 2015). SOASP has the intent to increase quality of life during the course of the disease. Patients participating in the programme meet at group sessions and are taught about their disease, how to manage and cope with OA symptoms, and the rationale for exercising, by physiotherapists or occupational therapists. Participants are also offered individually adapted physical exercises, to be carried out in group training sessions or individually. The National Board of Health and Welfare of Sweden recommends non-surgical treatment options before listing OA patients for total joint replacement (Socialstyrelsen 2014). According to data from the Swedish Hip Arthroplasty Register 2015, the proportion of hip OA patients that have visited a physiotherapist (47–89%) or taken part in the SOASP (10–63%) prior to THR, varies widely between different regions in Sweden (Kärrholm et al. 2016).

We investigated the influence of self-reported exposure to physiotherapy and/or patient education before THR on patient-reported outcomes 1 year postoperatively. In addition we explored demographic differences in the patient groups receiving or not receiving physiotherapy and/or patient education. 

## Patients and methods

This is an a posteriori study of observational routinely collected health data from the Swedish Hip Arthroplasty Register (SHAR) analyzing the influence of preoperative self-reported exposure to physiotherapy and/or patient education on patient-reported outcomes 1 year after THR.

### The Swedish Hip Arthroplasty Register (SHAR)

Data were obtained from the SHAR. This national joint registry has a coverage of all the hospitals performing hip replacements in the country and had a completeness of 98.3% of all total hip replacements performed in 2015 (Kärrholm et al. 2018). All data on primary THRs, including PROMs, are collected by the participating units, and entered into the register database using 2-factor authentication (Kärrholm et al. 2008).

### Patient-reported outcome measures in SHAR

Since 2002, SHAR has gathered PROMs from THR patients. In conjunction with the preoperative visit, patients are asked to complete a short survey (paper and digital version available according to the unit’s preference) including the EuroQol 5 dimensions (EQ-5D), a hip pain visual analogue scale (pain VAS), and self-reported Charnley classification. At 1-year postoperative follow-up, the same pen-and-paper survey is sent by ordinary mail with the addition of a satisfaction item on a VAS. The SHAR PROMS program has been described in detail previously (Rolfson et al. 2011).

EQ-5D measures health-related quality of life and consists of 2 parts. For the first part, we used the British value set to calculate the EQ-5D index, which ranges from –0.59 to 1.0, where 1.0 corresponds to perfect health and negative results to a state worse than death (Dolan and Roberts 2002). In the second part, the patient estimates his or her current health status on a 100-degree scale, where 0 corresponds to worst imaginable health and 100 to best imaginable health.

With pain VAS, the patient estimates his or her current pain on a visual 100-degree scale, where 0 corresponds to no pain and 100 to worst imaginable pain.

Satisfaction VAS measures patient satisfaction with the outcome of surgery on a visual 100-degree scale, where 0 corresponds to a completely satisfied patient and 100 to an unsatisfied patient.

In 2012, 2 questions (yes/no) were added to the preoperative survey: (1) “Have you been to see a physiotherapist for your hip during the period of hip problems?” and (2) “Have you taken part in a so-called SOASP (may have been many years before the operation for a shorter period of time) during the period of hip problems.” The response rate in 2014 was 85% for preoperative PROMs and 90% for the 1-year follow-up postoperative PROMs (Garellick et al. 2015).

### Patient selection

Data retrieved from SHAR covered hip OA (ICD-10 codes M16*) patients who had undergone primary THR surgery (NOMESCO codes NFB29, 39, and 49) between 2012 and 2015, with the years covering all available data including physiotherapy, patient education, and 1-year postoperative PROMs. Data retrieval was done in March 2018 and included age, sex, surgery side, first or second surgery, BMI, ASA class, Charnley class, incision, fixation, patient education, and physiotherapy. In addition, all PROMs collected preoperatively and/or postoperatively (pain VAS, EQ-5D and EQ VAS, and postoperative satisfaction VAS) were included.

We selected the surgeries where patients had their first primary THR, i.e., they had no previous hip replacement of their contralateral hip. Additional selection criteria were applied to exclude patients missing data (BMI, ASA class, incision, type of fixation, patient education, physiotherapy, preoperative and 1-year postoperative PROMs).

Patients with extreme values (BMI < 15 and BMI > 50) were excluded since these were probably erroneously recorded.

### Statistics

The software SPSS statistics version 25 (IBM Corp, Armonk, NY, USA) was used for all statistical analyses. The null hypothesis was rejected when p < 0.05.

Continuous variables were compared by using paired t-test. Categorical variables were analyzed by conducting Pearson’s chi-square tests to check for statistical significance between the 2 groups. 95% confidence intervals (CI) were calculated when appropriate.

### Linear regression analysis

The linear regression analyses were made by using a generalized linear model. A 95% confidence interval was used. The dependent variables used in the model were the postoperative PROMs: pain VAS, EQ-5D index, EQ VAS, and satisfaction VAS. The independent variables included were: age, sex, BMI, ASA class, Charnley class, incision, fixation, patient education, and physiotherapy. Also, for the preoperative pain VAS, EQ-5D index, and EQ VAS the corresponding preoperative variables were used as independent variables in the models.

### Non-respondent analysis

3 different non-respondent analyses were performed. First, patients with missing data on preoperative PROMs, physiotherapy, and patient education were compared with the patients included in the current analysis (study group). Second, cases excluded due to patients having second hip surgery and, third, the group with missing postoperative PROMs were compared with the study group. The method used was ANOVA with post-hoc Tukey with continuous variables and Pearson’s chi-square test with categorical variables.

### Ethics, funding, and potential conflicts of interest

This study is a part of a larger research project which has been reviewed and approved by the Regional Ethical Review Board in Gothenburg (2014-04-09, 271-14). The study was partly financed by grants from the Swedish state under the agreement between the Swedish government and the county councils, the ALF-agreement (ALFGBG–522591). The authors declare no conflicts of interest.

## Results

### Demographics (Table 1)

**Figure 1.**
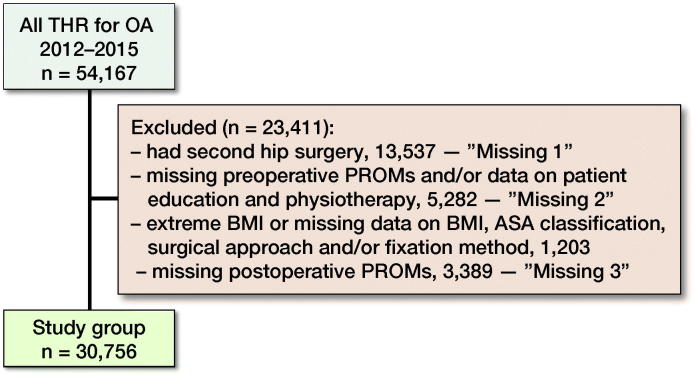
The flowchart describes the selection of patients. As defined here, excluded patients form 3 groups: missing 1, missing 2, and missing 3, further investigated in the missing data/non-respondent analysis (see Appendix). Abbreviations: THR = total hip replacement, OA = osteoarthritis, BMI = body mass index, ASA = American Association of Anesthesiologists’ classification.

Of all the 54,167 cases obtained from SHAR, 30,756 (59%) met the selection criteria and were included in the regression analyses (Figure 1). Of these, 71% reported exposure to physiotherapy, patient education or both, prior to surgery. Among the study group, 68% reported exposure to physiotherapy and 27% reported exposure to patient education, prior to surgery. At baseline, patients exposed to PT/SOASP had a statistically significantly lower age, BMI, preoperative EQ-5D index, and preoperative EQ-VAS, but higher preoperative pain VAS when compared with patients not exposed, on average. The PT/SOASP group also had a higher proportion of women, ASA I–II, and Charnley class A, and a lower proportion of cemented surgery.

**Figure 2.**
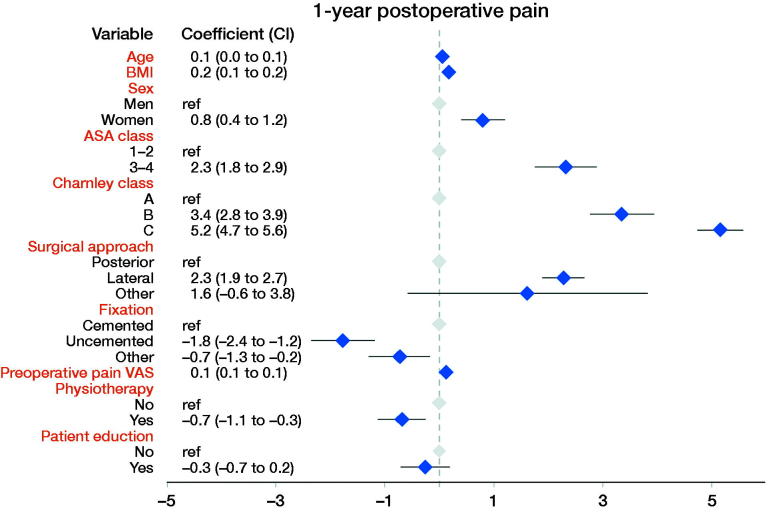
Linear regression results with the dependent variable pain VAS 1 year postoperatively.

**Figure 3.**
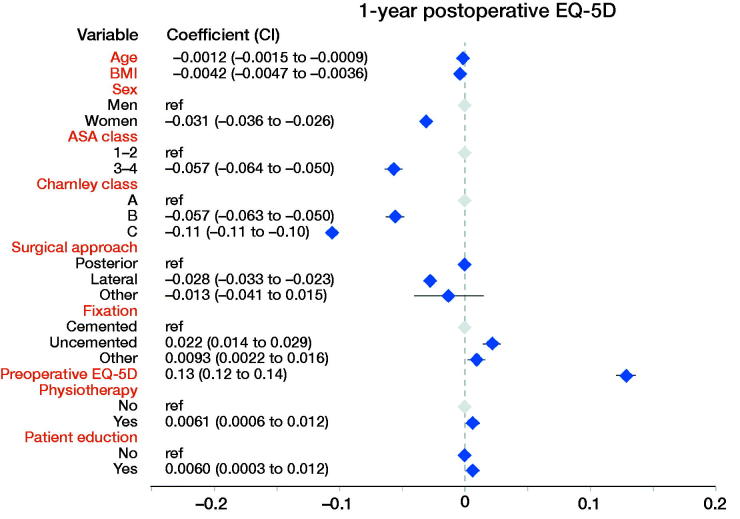
Linear regression results with the dependent variable EQ-5D 1 year postoperatively.

**Figure 4.**
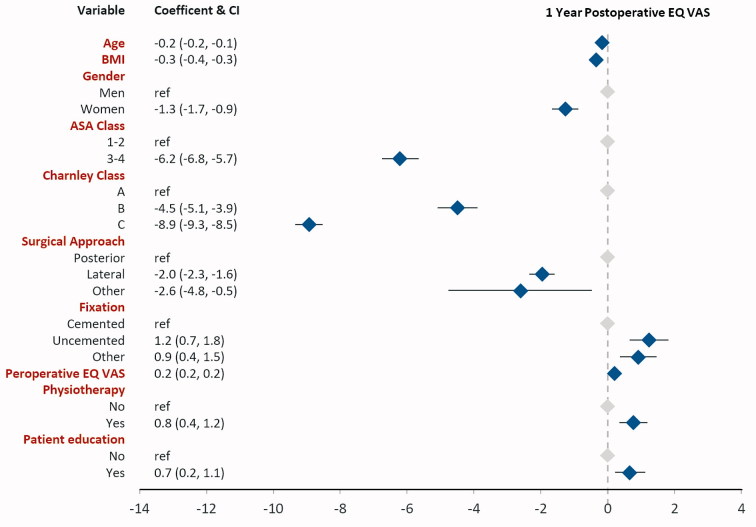
Linear regression results with the dependent variable EQ VAS 1 year postoperatively.

**Figure 5.**
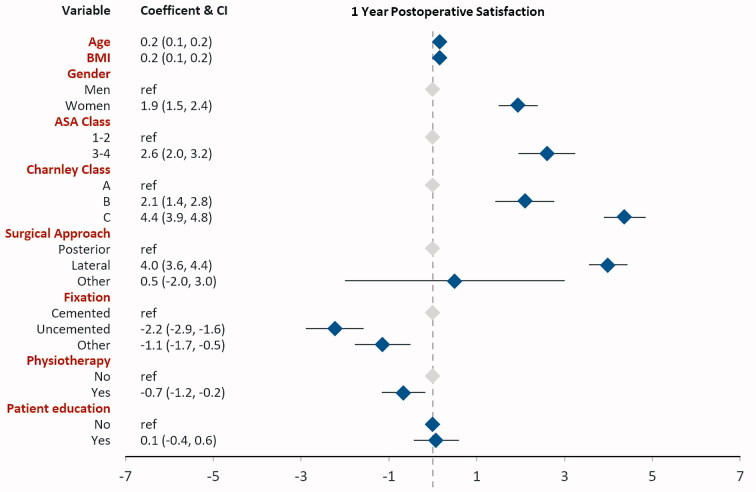
Linear regression results with the dependent variable satisfaction 1 year postoperatively.

### Linear regression analysis (Figures 2–5)

Physiotherapy was associated with 0.7 units lower pain VAS (CI –0.3, –1.1), 0.01 units higher EQ-5D index (CI 0.00, 0.01), 0.8 units higher EQ VAS (CI 0.4, 1.2), and 0.7 units lower (= better) score on the satisfaction VAS (CI –1.2, –0.2) postoperatively.

Self-reported patient education was associated with better EQ-5D index and EQ VAS. Patient education was associated with 0.006 units higher EQ-5D (0.01, CI 0.00, 0.01) and 0.7 units higher EQ VAS (0.7, CI 0.2, 1.1). Patient education did not influence pain VAS (–0.3, CI –0.7, 0.2) or satisfaction VAS (0.1, CI –0.4, 0.6) postoperatively.

### Non-respondent and missing data analysis

Compared with the study group, patients excluded due to having THR on their second hip had on average higher age, higher BMI, and higher preoperative pain VAS. They also had a higher proportion of females, Charnley class A, and cemented fixation, but a lower proportion of ASA I–II. Compared with the study group, patients with missing data on preoperative PROMs, physiotherapy, or patient education had on average higher age. They also had a higher proportion of cemented fixation as well as a lower proportion of ASA I–II and posterior incision. As for patients missing postoperative PROMs, they had on average lower age, higher BMI, higher preoperative pain VAS, lower EQ-5D index, and lower EQ-VAS, compared with the study group (Table 2, see Supplementary data).

## Discussion

In this study based on a national registry, patients visiting a physiotherapist at some point during the course of their disease had statistically significantly better 1-year postoperative PROMs. However, the positive influence was only on a par with or below the smallest factors the model was adjusted for, making the clinical relevance of the results uncertain.

For patients with hip OA, physiotherapy in the form of supervised exercise has been shown to reduce pain and improve function (Hernandez-Molina et al. 2008, Fransen et al. 2014). A few authors have also reported beneficial preoperative effects of supervised exercise for patients awaiting surgery (Wallis et al. 2011, Gill et al. 2013). A recent meta-analysis by Moyer et al. (2017) evaluating postoperative effects after preoperative supervised exercise concluded that there were improvements for pain, function, and length of stay. One of the included studies (Rooks et al. 2006) did not show any positive postoperative effects on PROMs and another RCT found no effects lasting past 6 weeks postoperatively following total knee and hip arthroplasty (Villadsen et al. 2014). There is 1 small RCT with 21 participants that showed better postoperative PROMs for THR patients following preoperative supervised exercise (Ferrara et al. 2008). In that study, patients in the exercise group had a statistically significantly (0.97 points) lower pain VAS (scale 0–10) 3 months postoperatively compared with controls, though also had a non-significant lower pain VAS of 0.62 at baseline. Compared with our postoperative VAS difference of 0.69 (scale 0–100), their result was more than 10 times larger.

Our study shows a statistically significant positive association with health-related quality of life as measured with EQ-5D and EQ VAS but not for pain and satisfaction in patients participating in preoperative patient education. There is a lack of larger RCTs (Wang et al. 2016) investigating the role of patient education during the course of the disease in postoperative outcomes. A few review articles (Ibrahim et al. 2013, McDonald et al. 2014, Aydin et al. 2015) have tried to analyze association between surgery-oriented preoperative patient education and postoperative outcomes with only 1 (Ibrahim et al. 2013) concluding that preoperative patient education resulted in better postoperative PROMs. The contradictory findings in the literature are partly reflected by our findings with a slight improvement in quality of life but no effect on pain or satisfaction in patients operated with THR following preoperative patient education.

There are limitations in this study. First, the physiotherapy interventions were not defined. Some patients reporting they have visited a physiotherapist might have received only non-exercise-based treatment, which potentially may reduce the association we found between physiotherapy and postoperative PROMs. The recommended physiotherapy-based treatment by the Swedish National Board of Health Welfare is long-term exercise (Socialstyrelsen 2012). However, the patients might have visited their physiotherapist before those recommendations were published and/or before receiving other interventions. In addition, we are not aware of how quickly and to what degree those recommendations have been implemented in Sweden.

Second, we do not know when the patients received their physiotherapy interventions, how regularly, to what intensity, or their compliance. This could affect our results. While there is a lack of validated evidence of preoperative exercise-based before joint replacement (Hoogeboom et al. 2012), the RCT in the field that has seen postoperative effects from preoperative physiotherapy has administered the interventions within a month from surgery and 5 times a week (Ferrara et al. 2008). The recruitment rate for physiotherapy within weeks before total joint replacement can be as low as 12% (Rooks et al. 2006) or 34% (Hoogeboom et al. 2010), with difficult transportation to the sessions a common complaint (Rooks et al. 2006, Hoogeboom et al. 2010). As 68% of the patients in our study group had answered “yes” to having been exposed to physiotherapy interventions, it is more likely that they have been exposed during earlier stages of the disease, with the intent of reducing OA symptoms.

The third limitation is the lack of information regarding to what degree patients have taken part in rehabilitation following THR. Geographic areas that have a higher availability of physiotherapy and SOASP might also have different availability of postoperative rehabilitation, potentially affecting our results.

The fourth limitation pertains to the demographic differences between the no PT/SOASP group and PT/SOASP group. 6 preoperative variables were favorable for the PT/SOASP group according to the factors’ coefficients on our regression analysis: age, BMI, ASA I–II, Charnley class, incision, and fixation. 4 preoperative variables were favorable for the no PT/SOASP group: sex, preoperative pain VAS, EQ-5D index, and EQ VAS. Though all those factors are adjusted for in the linear regression model, there is still a risk of having the results derive from confounding factors not accounted for in the modelling. Finally, there is also always a risk, when excluding patients, that the results do not represent the reality. The 3 excluded groups had worse baseline variables compared with the analysis group, predicting worse postoperative PROMs. If the patients in the excluded groups had had complete data and had been included in the analysis group, we would probably have seen overall worse postoperative PROMs. How the factors in the linear regression models would have been affected is, however, hard to forecast.

While much of the earlier focus has been on education and physiotherapy in close proximity to THR, our study indicates that interventions at some point during the course of OA have a positive influence on PROMs after surgery. Due to this study being observational, we cannot establish the causal relationships. Although earlier studies have not demonstrated lasting effects of physiotherapy post-THR, the influence observed in our study may be explained by increased compliance with supervised exercise after surgery. In OA patients without joint replacement, a previous systematic review article demonstrated the effect of supervised exercise lasting past 6 months with additional “booster” sessions with physiotherapists (Pisters et al. 2007). This could possibly be translatable for patients who have undergone THR.

Further studies with more specific questions of supervised exercises before and after surgery could increase our understanding. Larger RCTs further exploring specific preoperative exercise interventions and their effect postoperatively are also needed.

In summary, our study indicates that exposure to physiotherapy at some point during the course of OA has a small positive influence on 1-year postoperative PROMs after THR. Due to demographic differences, and uncertainties regarding the type of physiotherapy interventions and time frame, the clinical relevance of this small influence is uncertain. Therefore, the results should be interpreted with care. Further research is needed with more specific and demarcated physiotherapy interventions.

### Supplementary data

Table 2 is available as supplementary data in the online version of this article, http://dx.doi.org/10.1080/17453674.2019.1605669

The authors would like to thank all the surgeons and secretaries reporting to and coordinators maintaining the high quality and integrity of data being reported to the Swedish Hip Arthroplasty register.

CT, OR, and MM conceived and planned the study. CT performed statistical analyses. CT drafted the manuscript with subsequent substantial inputs from all co-authors.

*Acta* thanks Allan Abbot and Siri Bjørgen Wintherfor help with peer review of this study.

**Table 1.** Demographics

		Physiotherapy and/or patient education		
Variable	Study group	No	Yes	p-value ^a^
Total numbers	30,756	9,040	21,716	
Age ^b^	68 (9.9)	70 (10)	68 (9.7)	< 0.01
Female ^c^	17,127 (56)	4,250 (47)	12,877 (59)	< 0.01
BMI ^b^	27.3 (4.3)	27.4 (4.4)	27.2 (4.3)	< 0.01
ASA I–II ^c^	26,315 (86)	7,356 (81)	18,959 (87)	< 0.01
Charnley class ^c^				< 0.01
A	14,946 (49)	4,372 (49)	10,574 (49)	
B	4,125 (13)	1,129 (13)	2,996 (14)	
C	11,685 (38)	3,539 (39)	8,146 (38)	
Incision ^c^				0.4
Posterior	16,316 (53)	4,743 (53)	11,573 (53)	
Lateral	14,205 (46)	4,227 (47)	9,978 (46)	
Other	235 (0.8)	70 (0.8)	165 (0.8)	
Fixation ^c^				< 0.01
Cemented	19,339 (63)	5,967 (66)	13,372 (62)	
Uncemented	6,165 (20)	1,673 (19)	4,492 (21)	
Other	5,252 (17)	1,400 (16)	3,852 (18)	
Pain VAS ^b^	63.2 (15.3)	62.7 (16.3)	63.4 (14.9)	< 0.01
EQ-5D index ^b^	0.42 (0.3)	0.43 (0.3)	0.42 (0.3)	< 0.01
EQ VAS ^b^	57.9 (22.1)	59.0 (21.8)	57.5 (22.2)	< 0.01

**^a^**A 2-column Pearson’s chi-square test was used on the categorical variables. An independent sample t-test was used on the continuous variables.

**^b^**Continuous variables, presented with frequency (standard deviation).

**^c^**Categorical variables, presented with frequency (percentage).

## Supplementary Material

Supplemental Material
