# Supplementary material for: Minor influence of patient education and physiotherapy interventions before total hip replacement on patient-reported outcomes: an observational study of 30,756 patients in the Swedish Hip Arthroplasty Register
Source: Acta Orthop. 2019 Apr 17;90(4):306–11. doi: 10.1080/17453674.2019.1605669 (PMC6718188; doi:10.1080/17453674.2019.1605669)
Supplement: Supplemental Material [file IORT_A_1605669_SM7495.pdf]

Supplementary data

Table 2. Non-respondent analysis

Variable	Study group	Non-respondent group Missing 1	Missing 2	Missing 3	P-value: Study group versus [a] Missing 1	Missing 2	Missing 3
Total numbers	30,756	13,537	5,282	3,389			
Age	69 (10)	70 (9.7)	69 (11)	66 (12)	< 0.01	< 0.01	< 0.01
Female	17,127 (56)	7,954 (59)	2,973 (56)	1,788 (53)	< 0.01	0.4	0.01
BMI	27 (4.3)	28 (5.0)	27 (4.9)	28 (4.7)	< 0.01	1.00	< 0.01
ASA I–II	26,315 (86)	11,016 (83)	4,085 (79)	2,726 (80)	< 0.01	< 0.01	< 0.01
Charnley class					< 0.01	–	< 0.01
A	14,946 (49)	6,257 (54)	–	1,477 (44)			
B	4,125 (13)	882 (7.6)	–	456 (14)			
C	11,685 (38)	4,512 (39)	–	1,456 (43)			
Incision					0.1	< 0.01	0.1
Posterior	16,316 (53)	7,323 (53)	2,791 (53)	1,819 (54)			
Lateral	14,205 (46)	6,121 (45)	2,479 (47)	1,555 (46)			
Other	235 (0.8)	93 (0.7)	12 (0.2)	15 (0.4)			
Fixation					< 0.01	< 0.01	< 0.01
Cemented	19,339 (63)	8,931 (66)	3,329 (63)	1,737 (51)			
Uncemented	6,165 (20)	2,523 (19)	1,141 (22)	898 (27)			
Other	5,252 (17)	2,079 (15)	808 (15)	754 (22)			
Preop pain VAS	63.2 (15.3)	63.7 (15.3)	–	65.3 (15.7)	0.02	–	< 0.01
Preop EQ-5D index	0.42 (0.31)	0.43 (0.31)	–	0.36 (0.33)	0.08	–	< 0.01
Preop EQ VAS	57.9 (22.1)	58.1 (22.2)	–	54.7 (22.8)	0.9	–	< 0.01

[a] All missing groups were compared with the "study group." ANOVA post-hoc Tukey was used on the variables age and BMI. Independent sample t-test was used on preop pain VAS, EQ-5D index, and EQ VAS. Chi-square was used on ASA 1 and 2, Charnley class, incision, and fixation.
Missing 1: Patients excluded due to having second primary hip surgery.
Missing 2: Patients excluded due to missing preoperative PROMs and/or missing data on physiotherapy and/or SOASP.
Missing 3: Patients excluded due to missing postoperative PROMs.
